# Knockout of M-LP/Mpv17L, a newly identified atypical PDE, induces physiological afferent cardiac hypertrophy in mice

**DOI:** 10.1007/s11248-023-00373-7

**Published:** 2023-10-18

**Authors:** Reiko Iida, Misuzu Ueki, Toshihiro Yasuda

**Affiliations:** 1 Molecular Neuroscience Unit, School of Medical Sciences, University of Fukui, Fukui, 910-1193 Japan; 2 Organization for Life Science Advancement Programs, University of Fukui, Fukui, 910-1193 Japan

**Keywords:** Mpv17-like protein, Cyclic nucleotide phosphodiesterase, cAMP/PKA signaling, Cardiac hypertrophy

## Abstract

M-LP/Mpv17L (Mpv17-like protein) is an atypical cyclic nucleotide phosphodiesterase (PDE) without the molecular structure characteristic of the PDE family. Deficiency of M-LP/Mpv17L in mice has been found to result in development of β-cell hyperplasia and improved glucose tolerance. Here, we report another phenotype observed in *M-LP/Mpv17L*-knockout (KO) mice: afferent cardiac hypertrophy. Although the hearts of *M-LP/Mpv17L*-KO mice did not differ in size from those of wild-type mice, there was marked narrowing of the left ventricular lumen and thickening of the ventricular wall. The diameter and cross-sectional area of cardiomyocytes in 8-month-old *M-LP/Mpv17L*-KO mice were increased 1.16-fold and 1.35-fold, respectively, relative to control mice, but showed no obvious abnormalities of cell structure, fibrosis or impaired cardiac function. In 80-day-old KO mice, the expression of hypertrophic marker genes, *brain natriuretic peptide* (*BNF*), *actin alpha cardiac muscle 1* (*ACTC1*) and *actin alpha 1 skeletal muscle* (*ACTA1*), as well as the Wnt/β-catenin pathway target genes, *lymphoid enhancer-binding factor-1* (*LEF1*), *axis inhibition protein 2* (*AXIN2*) and *transcription factor 7* (*TCF7*), was significantly up-regulated relative to control mice, whereas fibrosis-related genes such as *fibronectin 1* (*FN1*) and *connective tissue growth factor* (*CTGF*) were down-regulated. Western blot analysis revealed increased phosphorylation of molecules downstream of the cAMP/PKA signaling pathway, such as β-catenin, ryanodine receptor 2 (RyR2), phospholamban (PLN) and troponin I (cTnI), as well as members of the MEK1-ERK1/2 signaling pathway, which is strongly involved in afferent cardiac hypertrophy. Taken together, these findings indicate that M-LP/Mpv17L is one of the PDEs actively functioning in the heart and that deficiency of M-LP/Mpv17L in mice promotes physiological cardiac hypertrophy.

**Supplementary Information:**

The online version contains supplementary material available at 10.1007/s11248-023-00373-7.

## Introduction

Intracellular signaling in cardiac disease has been intensively analyzed since the 1990s using cells and/or gene-modified animals. Cyclic nucleotides are second messengers involved in many physiological and pathophysiological processes. Cyclic nucleotide phosphodiesterases (PDEs) are responsible for the neurohormonal control of cardiac function by degrading cAMP and cGMP and are heavily implicated in cardiac disease (Bobin et al. 2016). There are 11 known PDE families, encoded by 21 genes, which generate nearly 100 isoforms (Omori and Kotera 2007).　Each PDE exhibits a unique tissue expression pattern, some PDEs localizing to the cytoplasm and others to intracellular organelles such as the nucleus, endoplasmic reticulum, Golgi and mitochondria and modulating their levels within microdomains, thereby forming local cyclic nucleotide signals. In the heart, in addition to PDE3 and PDE4, which predominate in the regulation of myocardial excitation and contraction via cAMP degradation, other PDE families, including PDE1, 2, 5, 9 and 10, each play a unique role in local cyclic nucleotide signaling pathways. Recent findings suggest that changes in PDE activity and/or localization may be closely related to the progression of cardiac disease, and that activators and inhibitors for specific PDEs might have therapeutic potential.

M-LP/Mpv17L (Mpv17-like protein) was originally discovered in mouse kidney during screening for genes with age-dependent expression (Iida et al. 2000, 2001).　M-LP shows high sequence homology to Mpv17 (Spinazzola et al. 2006), the product of the gene responsible for mitochondrial DNA depletion syndrome. In kidney and liver cells, suppression of M-LP/Mpv17L has been shown to increase mtDNA damage. We have recently revealed that M-LP/Mpv17L is an atypical PDE actually functioning in cells despite lacking the conserved catalytic region and other structural motifs characteristic of the PDE family (Iida et al. 2020). *M-LP/Mpv17L*-knockout (KO) mice survive to adulthood and develop pancreatic β-cell hyperplasia. Pancreatic islets from *M-LP/Mpv17L*-KO mice show up-regulation of *lymphoid enhancer-binding factor-1* (*LEF1*) and *transcription factor 7* (*TCF7*), which are major nuclear effectors of the Wnt/β-catenin signalling pathway, and increased phosphorylation of β-catenin and glycogen synthase kinase-3 beta (GSK-3β), suggesting activation of the Wnt/β-catenin signaling pathway (Iida et al. 2022). Here, we report another phenotype of *M-LP/Mpv17L*-KO mice, afferent cardiac hypertrophy, and discuss the effects of M-LP/Mpv17L deficiency on cardiac growth.

## Results and discussion

### Age-dependent changes of M-LP/Mpv17L expression in the heart

**Fig. 1:**
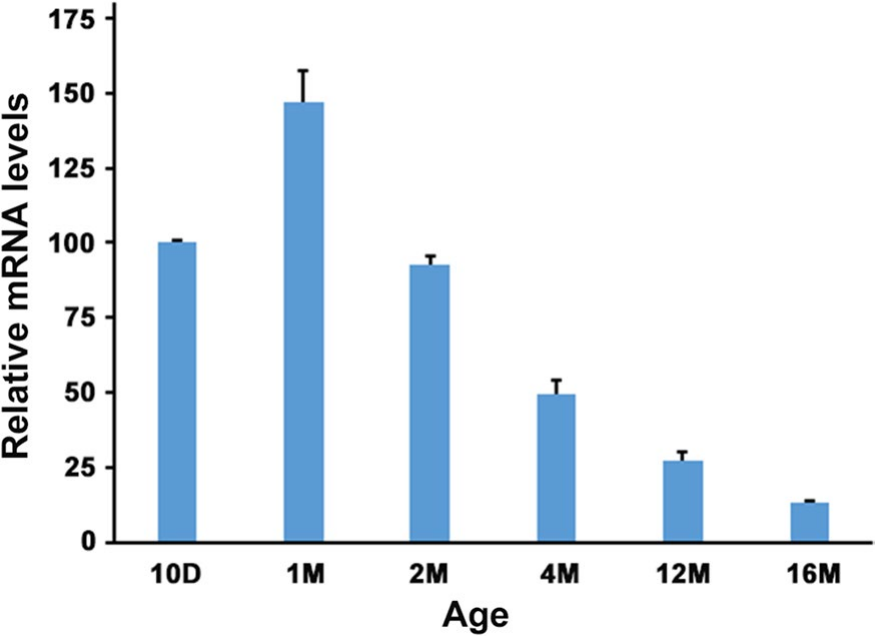
Levels of M-LP/Mpv17L mRNA in mouse hearts at various ages. mRNA expression was determined by Q-PCR using β-actin as an internal control. The results are expressed as ratios relative to the value for 10-day-old mice (n = 3)

First, we investigated whether M-LP/Mpv17L also shows age-dependent expression in the heart. Previous studies have shown that M-LP/Mpv17L is expressed in an age-dependent manner in the kidney (Iida et al. 2001) and pancreatic islets (Iida et al. 2022) of mice, expression beginning to increase around 3 weeks of age, peaking in adulthood and then declining. In the heart of wild-type (WT) mice, M-LP/Mpv17L expression was already high at 10 days of age, when expression is low in the kidney and pancreatic islets. It then peaked at around one month of age and decreased thereafter, meaning that the start and peak of expression shifted towards a younger age in comparison with the expression in kidney and pancreatic islets (Fig. 1).

We have reported previously that hyperplasia of pancreatic β-cells observed in *M-LP/Mpv17L*-KO mice might be caused by activation of the Wnt/β-catenin pathway. The Wnt/β-catenin pathway is involved in cell proliferation and differentiation and regulates the formation of various organs, being essential for development of the pancreatic endocrine system (Schinner et al. 2008). In the islets of *M-LP/Mpv17L*-KO mice, this pathway was activated through a series of steps, starting with increased cAMP levels and culminating in enhanced nuclear translocation of β-catenin. In other words, expression of M-LP/Mpv17L acts to inhibit cell proliferation through inactivation of the Wnt/β-catenin pathway. It is therefore plausible that M-LP expression is low in the growing mouse pancreas, where endocrine cells are actively proliferating, and high in the non-proliferating adult pancreas. On the other hand, the regulation of heart size is known to be biphasic: prenatally, cardiac growth is largely determined by cardiomyocyte proliferation, whereas after birth, total cell mass is regulated by signals controlling cardiac hypertrophy and proliferation of mouse cardiomyocytes seldom occurs later than 11 days after birth (Heallen et al. 2020; Alkass et al. 2015). Therefore, it is unlikely that M-LP/Mpv17L, which show relatively high expression in the heart at 10 days of age, would impede heart growth.

### M-LP/Mpv17L deficiency leads to physiological afferent cardiac hypertrophy in mice

**Fig. 2:**
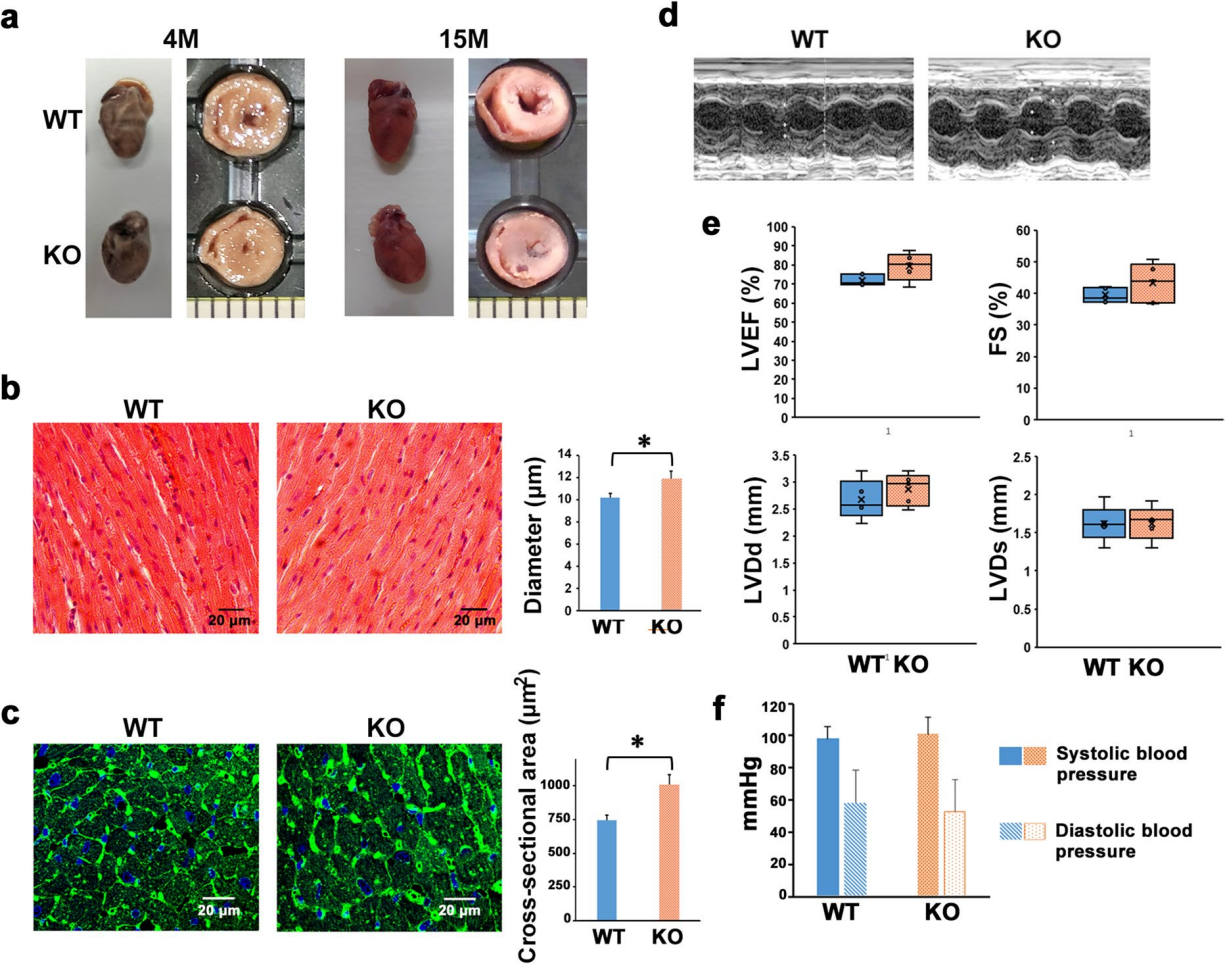
M-LP/Mpv17L deficiency leads to physiological afferent cardiac hypertrophy. a Gross morphology and cross-sections of representative hearts obtained from *M-LP/Mpv17L-*WT and -KO mice. b H&E staining of heart tissues from 8-month-old *M-LP/Mpv17L-*WT and -KO mice. Scale bars: 20 μm. Diameters of cardiomyocytes are shown as means ± SD (n = 10). *p < 0.05 c WGA staining of heart tissues from 8-month-old *M-LP/Mpv17L-*WT and -KO mice. Scale bars: 20 μm. Cross-sectional areas of cardiomyocytes are shown as means ± SD (n = 10). *p < 0.05 d Representative M-mode echocardiographic images of 14-month-old *M-LP/Mpv17L-*WT and -KO mice. e The echocardiographic parameters LVEF, FS, LVDd and LVDs of 14-month-old *M-LP/Mpv17L-*WT and -KO mice. Data are shown as means ± SD (n = 5). f Systolic and diastolic blood pressure of 16-month-old *M-LP/Mpv17L-*WT and -KO mice. Data are shown as means ± SD (n = 7)

In *M-LP/Mpv17L*-KO mice, gross morphometry showed that heart size did not differ from that in *M-LP/Mpv17L*-WT mice. However, the former showed afferent cardiac hypertrophy characterized by marked narrowing of the left ventricular lumen and thickening of the ventricular wall (Fig. 2a). Until at least 16 months of age, there was no early lethality or sign of heart failure. The diameter and cross-sectional area of cardiomyocytes in 8-month-old *M-LP/Mpv17L*-KO mice measured by hematoxylin and eosin (H&E) and wheatgerm agglutinin (WGA) staining, respectively (Fig. 2b, c), were 1.16-fold and 1.35-fold larger than those in *M-LP/Mpv17L*-WT mice. However, there was no obvious abnormality of cell structure or fibrosis. Echocardiography of 14-month-old mice revealed no significant differences between WT and KO mice in left ventricular ejection fraction (LVEF), fractional shortening (FS), left ventricular end-diastolic diameter (LVDd) or left ventricular end-systolic diameter (LVDs), but a trend for LVEF and FS – indicators of contractility – to be increased (Fig. 2d, e). Moreover, lack of any differences in blood pressure ruled out the possibility that chronic hypertension was the cause of the hypertrophy (Fig. 2f). Accordingly, it was considered that the deficiency of M-LP/Mpv17L had been the cause of the physiological afferent cardiac hypertrophy.

**Fig. 3:**
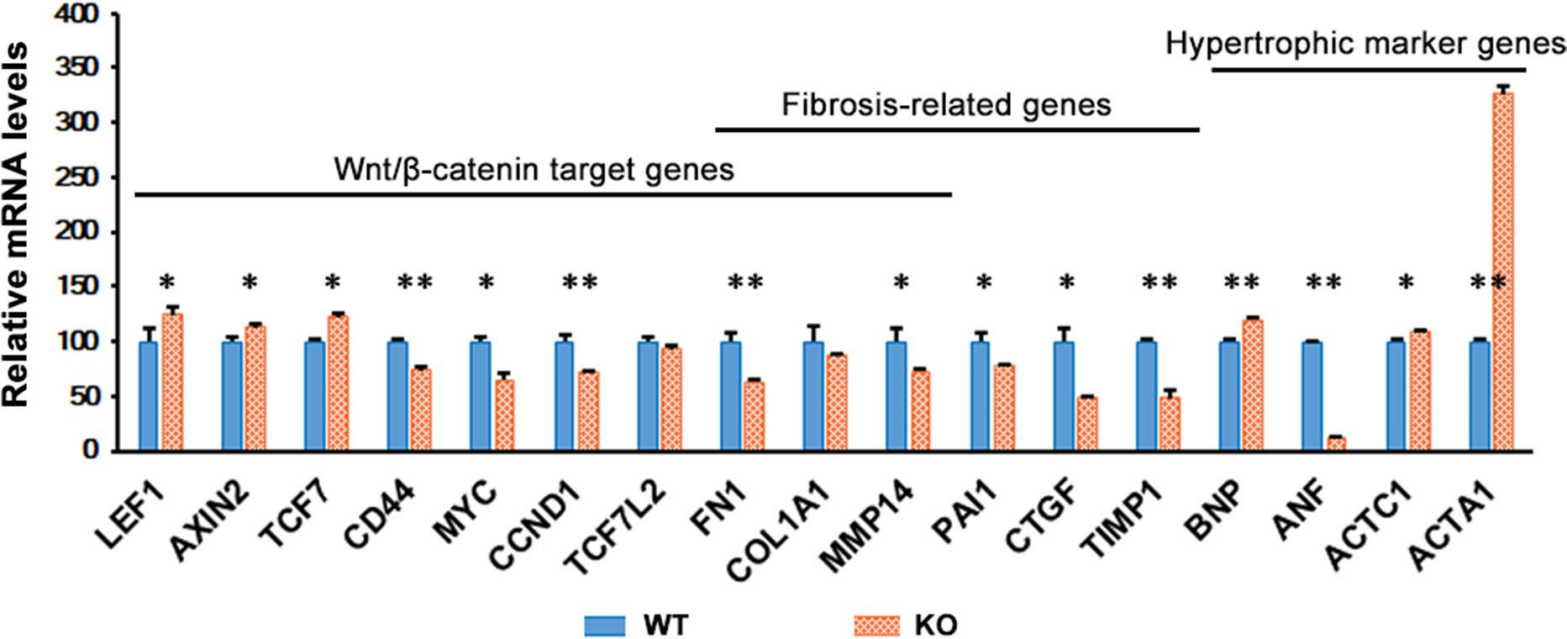
Transcriptomic analysis of hearts obtained from 80-day-old *M-LP/Mpv17L-*WT and -KO mice. Expression of mRNA was determined by Q-PCR using GAPDH as an internal control. The results are expressed as ratios relative to the value for *M-LP/Mpv17L*-WT mice (n = 3). *p < 0.05, **p < 0.005

To investigate the molecular mechanism responsible for the afferent cardiac hypertrophy observed in *M-LP/Mpv17L*-KO mice, we comparatively analyzed the expression of several genes downstream of the cAMP signaling pathway that have been reported to be involved in cardiac hypertrophy and/or myocardial fibrosis, using 80-day-old mice in which M-LP/Mpv17L is sufficiently expressed. As shown in Fig. 3, among the Wnt/β-catenin pathway target genes, the expression of *LEF1*, *axis inhibition protein 2* (*AXIN2*) and *TCF7*, which bind directly to β-catenin, was significantly increased in *M-LP/Mpv17L*-KO mice, while the expression of other Wnt/β-catenin pathway target genes and fibrosis-related genes, such as *fibronectin 1* (*FN1*) and *connective tissue growth factor* (*CTGF*), was significantly decreased. Expression of the hypertrophic marker genes, *brain natriuretic peptide* (*BNF*), *actin alpha cardiac muscle 1* (*ACTC1*) and *actin alpha 1 skeletal muscle* (*ACTA1*), was significantly increased, whereas that of *atrial natriuretic factor* (*ANF*) was significantly decreased; in particular expression of *ACTA1*, a major component of the contractile apparatus, was increased more than three-fold, while that of *ANF*, a natriuretic and vasorelaxant peptide, was decreased to less than one-fifth. These discordant changes in gene expression indicate that the hypertrophy response is regulated differentially in the cardiomyocytes of *M-LP/Mpv17L-*KO mice.

**Fig. 4:**
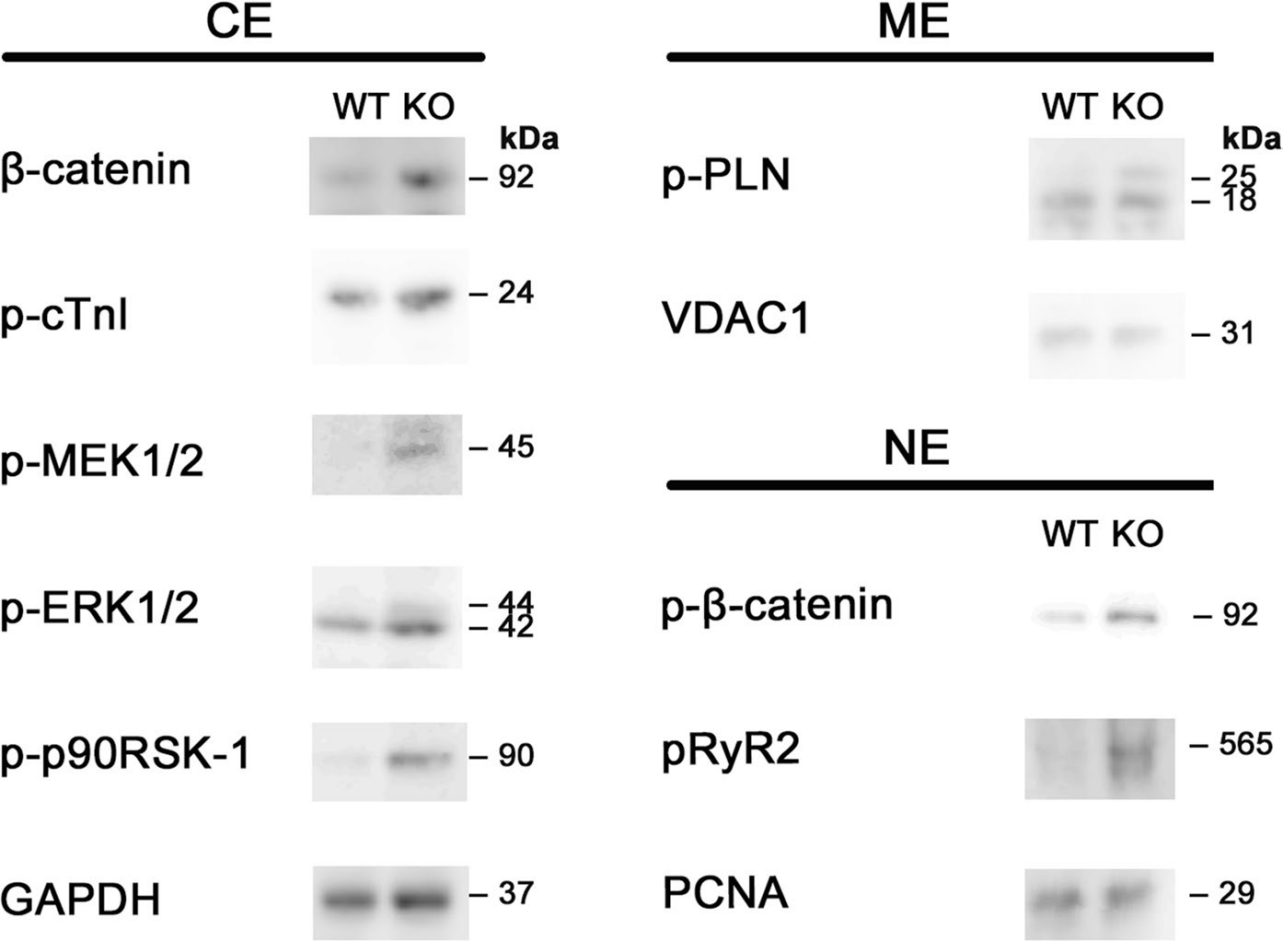
Western blot analysis of cytosolic, membrane and nuclear soluble extracts from 80-day-old *M-LP/Mpv17L-*WT and -KO mice. GAPDH, VDAC1 and PCNA were used as loading controls for cytosolic, membrane and nuclear protein, respectively. CE: cytosolic extract; ME: membrane extract; NE: nuclear soluble extract

Next, we examined changes in the phosphorylation status of molecules downstream of the cAMP/PKA signaling pathway, such as β-catenin, phospho-ryanodine receptor 2 (RyR2), phospholamban (PLN) and troponin I (cTnI), as well as members of the mitogen-activated protein kinase kinase 1 (MEK1)-extracellular signal-regulated kinase 1/2 (ERK1/2) signaling pathway, which has been strongly implicated in afferent cardiac hypertrophy (Fig. 4).

First, increased phosphorylation of Ser675 at the C-terminus and nuclear translocation of β-catenin were observed, consistent with the increased expression of several Wnt/β-catenin pathway target genes described above. The Wnt/β-catenin pathway is widely known to be an important regulator of cardiac development and growth. Furthermore, key factors in this pathway, such as GSK-3β, β-catenin and AXIN, are reportedly involved in many Wnt-independent cellular processes (Ni et al. 2023; Li et al. 2022). With regard to β-catenin, several studies have suggested that Wnt-independent stabilization and accumulation of β-catenin lead to the induction of cardiac hypertrophy. For example, overexpression of β-catenin was shown to induce myocardial hypertrophy (Haq et al. 2003; Ni et al. 2023), and cardiac hypertrophy occurring after transverse aortic constriction was found to be reduced in β-catenin heterozygous mice relative to wild-type mice (Qu et al. 2007). These experimental results support the possibility that one of the causes of cardiac hypertrophy observed in *M-LP/Mpv17L*-KO mice is stabilization of β-catenin, which begins upon activation of the cAMP/PKA pathway.

Secondly, it was shown that the phosphorylation levels of three molecules associated with excitation-contraction coupling (ECC) are elevated in *M-LP/Mpv17L*-KO mice: RyR2, which regulates the release of calcium ions from the sarcoplasmic reticulum; PLN, an inhibitor of sarcoplasmic reticulum Ca^2+^-ATPase; and cTnI, which is involved in muscle contraction. These changes in ECC-associated molecules indicate enhanced myocardial contractility and increased relaxation in KO mice. Increased PKA-mediated phosphorylation of RyR2 has also been reported in *PDE4B*- and *PDE4D*-KO mice (Mika et al. 2014; Lehnart et al. 2005). PDE4B is a component of the L-type Ca^2+^ channel (LTCC) complex and involved in regulating the LTCC current. In *PDE4B*-KO mice, the phosphorylation statuses of LTCC and RyR2 were altered, while PLN and cTnI were unaffected, suggesting that PDE4B is mainly involved in the regulation of local cAMP signaling near the sarcolemma (Mika et al. 2014). The structure and molecular size of M-LP/Mpv17L differ significantly from those of known PDEs, suggesting that the former acts in different microdomains within the cell and plays a specific role.

Thirdly, increased phosphorylation of successive kinases, MEK1/2 (MAPKK), ERK1/2 (MAPK) and 90-kDa ribosomal s6 kinase (p90RSK), was observed and activation of the prototypical MEK-ERK1/2 signaling pathway was suggested. The MEK-ERK1/2 signaling pathway is an important regulator of cardiac development and hypertrophy and is known to be activated in heart diseases such as hypertrophic and dilated cardiomyopathy (Wang 2007). Bueno et al. (2000) reported that mice overexpressing MEK1 in the heart exhibited ERK1/2 activation and marked afferent hypertrophy, but cardiac function was preserved and the hypertrophy was physiological without interstitial fibrosis. Their results are consistent with our finding that *M-LP/Mpv17L*-KO resulted in activation of the MEK1-ERK1/2 signaling pathway and, consequently, physiological cardiac hypertrophy. The results of the present study indicate that inhibitors of M-LP/Mpv17L may have potential as therapeutic agents for cardiac disease, and improved knowledge of the molecular biology of M-LP/Mpv17L and/or its association with disease will hopefully prove useful in the future.

## Materials and methods

### Mice

*M-LP/Mpv17L*-KO mice were generated on a C57BL/6N background according to our previous paper (Iida et al. 2022). The design of the animal experiment was approved by the Committee for Animal Experiments of the University of Fukui.

### Quantitative-PCR (Q-PCR) analysis

Mice were killed by carbon dioxide inhalation. Each animal’s heart was dissected, immediately placed in RNA protect tissue reagent (Qiagen, Chatsworth, CA) and then stored at -80 °C. Total RNA was extracted from frozen tissues with a RNeasy Mini kit (Qiagen), and cDNA was synthesized using a PrimeScript RT reagent kit with gDNA Eraser (Takara Bio, Shiga, Japan). Q-PCR was performed using the StepOne plus real-time PCR System (Applied Biosystems, Foster city, CA) and Power SYBR Green Master Mix (Applied Biosystems) in accordance with the manufacturer’s instructions. All PCR assays were performed at least three times. The primer sequences used for the Q-PCR analyses are summarized in Table S1.

### Histological analysis

The hearts were dissected out and fixed in 4% (w/v) paraformaldehyde phosphate buffer solution for 24 h, then embedded in paraffin for further histological analysis. H&E staining was used for direct microscopic examination. For WGA staining, paraffin sections were deparaffinized in Lemosol (Fujifilm Wako Cemicals, Osaka, Japan) and rehydrated via an ethanol/PBS series. The slides were then incubated with CF488A-conjugated WGA (1:200 in PBS) for 60 min, washed with PBS three times, and mounted in antifade reagent (SlowFade Diamond Antifade Mountant with DAPI, Molecular Probes). Fluorescence images were analyzed using a laser scanning confocal microscope (FV1200, Olympus, Tokyo, Japan). Diameter and cross-sectional area of cardiomyocytes in the ventricular septum were measured using the MicroStudio software package (Wraymer, Osaka, Japan).

### Assessment of cardiac function

Blood pressure measurements and characterization of cardiac function were performed using echocardiography equipment (ProSound SSD-α10, Aloka, Tokyo, Japan) and a blood pressure measuring device designed for mice (BP-98 A-L, Softron, Tokyo, Japan), respectively.

### Western blot analysis

Separation and preparation of cytosolic, membrane and nuclear protein extracts were performed using a Subcellular protein fractionation kit for tissues (Thermo Fisher Scientific, Waltham, MA). Western blotting was performed as described previously (Iida et al. 2020) using anti-glyceraldehyde-3-phosphate dehydrogenase (GAPDH, bs-2188R) antibody purchased from Bioss antibodies (Woburn, MA), anti-proliferating cell nuclear antigen (PCNA) antibody (MABE288) from EMD Millipore (Temecula, CA), anti-voltage-dependent anion-selective channel 1 (VDAC1) antibody (55259-1-AP) from Proteintech (Rosemont, IL), anti-β-catenin (#8480), anti-phospho-β-catenin Ser675 (#4176), anti-phospho-MEK1/2 Ser217/221 (#9154), anti-phospho-ERK1/2 Thr202/Tyr204 (#4370) and anti-phospho-p90RSK Ser380 (#11989) from Cell Signaling Technology (Danvers, MA), anti-phospho-RyR2 Ser2808 (BS4358) antibody from Bioworld technology (St Louis Park, MN), and anti-phospho-cTnl Ser23/Ser24 (#133976) and anti-phospho-PLN Ser16 (#15000) from Gene Tex (Irvine, CA). Additional antibodies including horseradish peroxidase (HRP)-conjugated anti-rabbit IgG antibody and HRP-conjugated anti-mouse IgG antibody were purchased from Thermo Fisher Scientific and Bio-Rad (Hercules, CA), respectively.

### Statistical analysis

All of the assays were performed at least 3 times, and the results were presented as mean ± S.D. Statistical analyses were performed by Student’s *t* test, at a significance level of p < 0.05.

### Electronic supplementary material

Below is the link to the electronic supplementary material.

Supplementary Material 1
