# Supplementary material for: Knockout of M-LP/Mpv17L, a newly identified atypical PDE, induces physiological afferent cardiac hypertrophy in mice
Source: Transgenic Res. 2023 Oct 18;32(6):575–82. doi: 10.1007/s11248-023-00373-7 (PMC10713670; doi:10.1007/s11248-023-00373-7)
Supplement: Supplementary file 1 — Supplementary Material 1 [file 11248_2023_373_MOESM1_ESM.docx]

Transgenic Research

Knockout of M-LP/Mpv17L, a newly identified atypical PDE, induces physiological afferent cardiac hypertrophy in mice

Reiko Iida • Misuzu Ueki • Toshihiro Yasuda

Reiko Iida (✉)

Molecular Neuroscience Unit, School of Medical Sciences, University of Fukui, Fukui 910-1193, Japan

e-mail: riida@u-fukui.ac.jp (R. Iida)

Misuzu Ueki

Molecular Neuroscience Unit, School of Medical Sciences, University of Fukui, Fukui 910-1193, Japan

Toshihiro Yasuda

Organization for Life Science Advancement Programs, University of Fukui, Fukui 910-1193, Japan

Supplementary Data

Table S1 　Primers used for Q-PCR analysis

| **Genes** | **Forward primer (5’-3’)** | **Reverse primer (5’-3’)** |
| --- | --- | --- |
| M-LP/Mpv17L | CCCACTAACGTGCTGCTCTA | TCTGATCGCATAGCACCTTG |
| LEF1 | CAAATAAAGTGCCCGTGGTG | TCGTCGCTGTAGGTGATGAG |
| AXIN2 | GCAGCTCAGCAAAAAGGGAAAT | TACATGGGGAGCACTGTCTCGT |
| TCF7 | GGACATCAGCCAGAAGCAAG | GGACAGGGGGTAGAGAGGAG |
| MYC | CTGTACCTCGTCCGATTCCA | ATCTTCTTGCTCTTCTTCAGAGTCG |
| TCF7L2 | GAAGGCGAGCAGGAGGAGAA | CCTCTTGGCCGCTTCTTCCA |
| CCND1 | GCGTACCCTGACACCAATCTC | CTCCTCTTCGCACTTCTGCTC |
| CD44 | CTCAGATTCCAGAATGGCTC | TCAGCTGTCATACACTGGTC |
| FN1 | CGAGGTGACAGAGACCACAA | CTGGAGTCAAGCCAGACACA |
| COL1A1 | ATCTCCTGGTGCTGATGGAC | ACCTTGTTTGCCAGGTTCAC |
| MMP14 | GCCCAAAAACCCCGCCTATG | ATTCCTCACCCGCCAGAACC |
| PAI1 | TGACAGTGGGAA GAGACGCC | GGGGGTAAGGAGGAGT TGCC |
| CTGF | ACTGTGTACGGAGCGTGACC | GTGCACACTCCGATCTTGCG |
| TIMP1 | ACAGCCTTCTGCAACTCGGA | CGCTGGTATAAGG TGGTCTCGT |
| BNP | TTGGGCTGTAACGCACTGAA | GGGGAAAGAGACCC AGGCAG |
| ANF | CCTGGGACCCCTCCGATAGA | AATCCTACCCCCGAAGCAGC |
| ACTA1 | AATGAGCGTTTCCGTTGCCC | ATGCGGTCAGCGATACCAGG |
| ACTC1 | TGAGCGCGGGTACTCCTTTG | AGTCTCTGGACAGCGGAAGC |
